# Rescaling quality of life values from discrete choice experiments for use as QALYs: a cautionary tale

**DOI:** 10.1186/1478-7954-6-6

**Published:** 2008-10-22

**Authors:** Terry N Flynn, Jordan J Louviere, Anthony AJ Marley, Joanna Coast, Tim J Peters

**Affiliations:** 1 Department of Social Medicine, University of Bristol, Canynge Hall, Whiteladies Road, Bristol BS8 2PR, UK; 2 Centre for the Study of Choice, University of Technology Sydney, City – Haymarket Campus, Broadway NSW 2007, Sydney, Australia; 3 Department of Psychology, University of Victoria, P.O. Box 3050, Victoria, B.C. V8W 3P5, Canada; 4 Department of Health Economics, Public Health Building, University of Birmingham, Birmingham B15 2TT, UK; 5 Department of Community Based Medicine, Department of Community Based Medicine, University of Bristol, 25 Belgrave Road, Bristol BS8 2AA, UK

## Abstract

**Background:**

Researchers are increasingly investigating the potential for ordinal tasks such as ranking and discrete choice experiments to estimate QALY health state values. However, the assumptions of random utility theory, which underpin the statistical models used to provide these estimates, have received insufficient attention. In particular, the assumptions made about the decisions between living states and the death state are not satisfied, at least for some people. Estimated values are likely to be incorrectly anchored with respect to death (zero) in such circumstances.

**Methods:**

Data from the Investigating Choice Experiments for the preferences of older people CAPability instrument (ICECAP) valuation exercise were analysed. The values (previously anchored to the worst possible state) were rescaled using an ordinal model proposed previously to estimate QALY-like values. Bootstrapping was conducted to vary artificially the proportion of people who conformed to the conventional random utility model underpinning the analyses.

**Results:**

Only 26% of respondents conformed unequivocally to the assumptions of conventional random utility theory. At least 14% of respondents unequivocally violated the assumptions. Varying the relative proportions of conforming respondents in sensitivity analyses led to large changes in the estimated QALY values, particularly for lower-valued states. As a result these values could be either positive (considered to be better than death) or negative (considered to be worse than death).

**Conclusion:**

Use of a statistical model such as conditional (multinomial) regression to anchor quality of life values from ordinal data to death is inappropriate in the presence of respondents who do not conform to the assumptions of conventional random utility theory. This is clearest when estimating values for that group of respondents observed in valuation samples who refuse to consider any living state to be worse than death: in such circumstances the model cannot be estimated. Only a valuation task requiring respondents to make choices in which both length and quality of life vary can produce estimates that properly reflect the preferences of all respondents.

## Background

The fundamental assumption underlying the quality-adjusted-life-year (QALY) model is that the estimated health state values should reflect the relative desirability of health states [1]. The aim of the QALY approach is to allow comparisons of interventions that affect life expectancy to those that affect quality of life. Several elicitation methods have been proposed to estimate QALY values reflecting people's preferences, with recent interest in methods requiring only ordinal respondent preferences [2-4]. Ranking responses such as stating that A is preferred to B (without reference to any numerical trade-offs) are cognitively easier than stating by *how much *A is preferred to B, and so make less stringent assumptions about the cognitive abilities required to compare options.

Discrete choice experiments (DCEs) are the most common type of ordinal task used in health services research to estimate utilities based on patient choices. DCEs can estimate health state values for two reasons. First, they are compatible with Lancaster's theory of value, which states that the total utility of a state can be decomposed into utilities of characteristics that describe it [5]; given an appropriate statistical design [6], utilities of alternative health states (profiles/specifications) allow one to infer utilities of attribute levels that describe them. Second, they are consistent with random utility theory (RUT), a well-tested theory of human decision-making [7,8]. RUT assumes that the total utility of a good/service can be expressed as the sum of two components, one fixed (systematic), and a second random (stochastic). If the random component is an independently and identically distributed (iid) extreme value type 1 (Gumbel) random variate, then the underlying choice process is consistent with McFadden's (1974) conditional (multinomial) logit model, and this model can be used to estimate the elements of the fixed component [7]. That is, the relative choice frequencies reveal the individuals' preferences (utilities), which can be estimated from the frequencies as a function of attribute levels. When the above model holds, and in particular when no choice probabilities equal 0 or 1, we refer to the model as the *conventional *random utility model.

Ranking [2,3] and best-worst experiments [4] (which elicit 'partial rankings') can be viewed as generalisations of DCEs [9] that can be used to obtain data to estimate utilities of individual attribute levels (and their interactions, given larger designs), or the utility of a profile. Two recent papers recognise and discuss the potential of RUT-based choice tasks to estimate such values. Salomon and McCabe *et al *proposed omitting length of life as a variable in the main valuation task [2,3], and asking respondents to choose between impaired living states and the death state. Specifically, respondents are required to rank several states (profiles) with death as one of the states. The idea is that by including the death state somewhere on the latent variable (continuum of health or quality of life), the difference between any given health (or quality of life) state and death on this variable can be estimated from the probability of choosing death over a particular (usually very bad) state. (Comparisons between the death state and good states are neither required nor generally meaningful due to nobody preferring death). This probability can be estimated at an individual level (if the respondent has made repeated choices) or at the sample level using the proportion of people choosing death.

This paper explores the model proposed by the above authors using data and results from the ICECAP (Investigating Choice Experiments for the Preferences of Older People-ICEPOP project – CAPability instrument) DCE. The ICECAP measure provides an index of capability for older people. It is not a QALY measure and is not intended to be such: the values do not represent the trade-offs people are willing to make between quantity and quality of life. Nevertheless, it is possible to treat the ICECAP data as if they were being used to generate QALYs, and thus to explore this model using the data generated by the valuation exercise for the ICECAP measure. Using these data, this paper shows that there is no reason to expect that the model estimates reflect the true quantity/quality trade-offs that the respondents will make. In fact, the utility estimates from such models will agree with those from time trade-off (TTO) or standard gamble (SG) tasks only by chance. Thus, the aims of this paper are to:

1) estimate QALY-like values from the ICECAP DCE data using the common model proposed by Salomon and McCabe *et al*; and

2) illustrate that their model estimates are sensitive to the proportion of people whose choices are consistent with conventional RUT.

The paper concludes with a research agenda.

## Methods

### Data – the ICECAP index of capability for older people

The ICECAP instrument was designed to give a set of general capability values for the UK population aged 65+ [10]. By focusing on general quality of life rather than health or health-related quality of life the measure can be used to compare across health and social care interventions. The measure has five attributes (attachment, security, role, enjoyment and control), each varied over four levels. A given state is described by the levels defining it: for instance state 22422 represents the state where every attribute takes level two, except role which takes level four (the most desirable level). An initial set of population level quality of life values was generated using preference-elicitation methods. Development of the attributes and estimation of these values (summarised in table 1) is described elsewhere [10,11].

**Table 1:** Terminology for attribute levels, and rescaled values, such that the absence of capability, state 11111, is equal to zero, and full capability, state 44444, is equal to one; level numbers appear in brackets.

**Attribute**	**Value**
**Attachment**	

(4) I can have all of the love and friendship that I want	0.2535

(3) I can have a lot of the love and friendship that I want	0.2325

(2) I can have a little of the love and friendship that I want	0.1340

(1) I cannot have any of the love and friendship that I want	-0.0128

**Security**	

(4) I can think about the future without any concern	0.1788

(3) I can think about the future with only a little concern	0.1071

(2) I can only think about the future with some concern	0.0661

(1) I can only think about the future with a lot of concern	0.0321

**Role**	

(4) I am able to do all of the things that make me feel valued	0.1923

(3) I am able to do many of the things that make me feel valued	0.1793

(2) I am able to do a few of the things that make me feel valued	0.1296

(1) I am unable to do any of the things that make me feel valued	0.0151

**Enjoyment**	

(4) I can have all of the enjoyment and pleasure that I want	0.1660

(3) I can have a lot of the enjoyment and pleasure that I want	0.1643

(2) I can have a little of the enjoyment and pleasure that I want	0.1185

(1) I cannot have any of the enjoyment and pleasure that I want	0.0168

**Control**	

(4) I am able to be completely independent	0.2094

(3) I am able to be independent in many things	0.1848

(2) I am able to be independent in a few things	0.1076

(1) I am unable to be at all independent	-0.0512

Adapted from Table 2 of the main ICECAP valuation paper with permission of the first author and Elsevier Publishing [11].

Sampling was restricted to those aged 65 and over, using the sampling frame of respondents to the Health Survey for England (HSE). The survey was interviewer-administered in respondents' homes. The main HSE survey (conducted 6–12 months earlier) provided additional data, such as: basic socio-demographic information; health; nature of locality and environment; social support; participation and contact with others; and general well-being.

The main valuation task was a best-worst scaling (BWS) exercise [12,13] that focuses on 'profiles' of 'attributes'; that is, respondents choose their most and least preferred attribute levels within each profile (quality of life state) they evaluate [4]. This minimises cognitive burden for respondents in this age group, important because traditional DCE tasks requiring the comparison of entire quality of life states can be cognitively difficult. However, two (simple) between-profile comparisons were included: 1) comparing each state with a respondent's own quality of life; and 2) comparing each state with death. The DCE with the latter choice included is described below.

### Design of the discrete choice experiment

Varying five attributes (K = 5), each with four levels (L = 4), meant that the total number of possible states was 4^5 ^= 1024. Due to practical constraints it was not possible to recruit enough respondents to estimate interactions. Therefore, two versions of an orthogonal main effects plan (OMEP) obtained from this website , as suggested by Street *et al *[14], were administered. An OMEP ensures that estimates of all (4 × 5 = 20) main effects are uncorrelated. This OMEP was used to make survey version 'A', and its foldover (levels 1 and 4, and levels 2 and 3, are swapped) was used to make survey version 'B'. Respondents were randomly allocated to receive version A or version B, and each version contained 16 quality of life states. For each of the 16 states, respondents were first asked if the quality of life state in question was at least as good as their own life. If they answered yes, it was assumed that they also considered the state to be better than immediate death. This assumption that nobody would rather die than continue living may be questionable but could not be tested, given concerns expressed by the ethics committee. If they answered no, they were asked if they considered the state in question to be 'a life worth living'.

### Random utility theory

As previously noted, RUT assumes that the utility of each state/profile has a fixed (systematic) component and a random (stochastic) component. Utilities are not known with certainty by researchers, hence are random variables. Thus, the probability that state *i *is chosen as best is equal to the probability that its utility is greater than the utility of every other state in a given choice set. Respondents are assumed to choose the state with the greatest utility. To operationalise the model one must make assumptions about the probability distribution of the random utility component.

### The conditional logit statistical model

The McFadden multinomial representation of random utility theory [7] (also set out by Holman and Marley in Luce and Suppes [15]) assumes that each random utility component is distributed as an iid extreme value type 1 (EV1 or Gumbel) random variate with zero mean and fixed variance [7,16]. This results in the distribution of the difference between (any) two states being logistic [17]. Thus, the conditional logit model assumes that each respondent perceives the difference between state 11111 (with every attribute taking its worst possible level) and death as a random variable from a logistic distribution. McCabe *et al *state that the odds of state *j *being chosen over state *k *is exp{*μ*_*j *_- *μ*_*k*_}, where *μ*_*i *_is the utility of state *i *[3], with the log odds estimating the utility difference of health states *j *and *k*. However, as is true of all limited (discrete value) dependent variable models, this model has an identification problem due to the error variance, or equivalently, the scale of the utility estimates being confounded with the model parameters. The importance of this is explained in the next section.

### Model estimation

The ICECAP DCE essentially asked respondents to choose between each state and death (subject to the assumption that a respondent would not prefer to die immediately); hence, one could use a regression model to estimate values that assign a zero value to death based on people's preferences, as proposed by McCabe *et al *and Salomon [2,3]. However, such model estimates from the ICECAP DCE are likely to be unreliable because the proportion of people who choose death as more preferred should tend to zero as quality of life states improve, yielding much less precise estimates. Indeed, if all respondents in a sample agreed that (for example) all states at least as good as 33333 (a state where each attribute has level 3) were worth living, one could not estimate the additional utility of level 4 compared with level 3 for any attribute. (As noted above, a ranking exercise involving inter-profile comparisons would not suffer from this limitation but such an exercise would not have been possible with this sample of respondents.) Another issue is that the assumption of constant error variance within and between respondents in the DCE is unlikely to be true because people are likely to be more consistent in their preferences as the quality of life state on offer becomes more or less attractive. In other words, the variance of the random utility term is likely to be small for very poor (good) states, reflecting general agreement that such states are highly unattractive (unattractive), but larger for intermediate states, reflecting disagreement on the relative value assigned to these. For these two reasons, choice consistency is likely to be higher for very attractive or unattractive states, and lower for states in between.

Problems of inconsistent choices and low precision are less likely in conventional ranking studies like those of McCabe *et al *and Salomon which compare health states with each other (rather than death) [2,3]. Therefore, to minimise such problems here, the full set of anchored values was not estimated directly from the DCE. Instead, the final values from Coast *et al *[11] which are population-level best-worst estimates of quality of life (capability) anchored such that state 11111 has zero value – were rescaled using the DCE estimates of two quality of life states. The importance of using a minimum of two states from the DCE to rescale will become clear from consideration of the random utility component. As noted earlier, estimates in all limited dependent variable models are confounded with the error variance [18]; thus, the estimate of the utility of (for example) state 11111 relative to death is actually its utility *divided by the standard deviation of the error distribution*. So, any particular odds ratio (for 11111 relative to death) is consistent with a large (small) difference in utility divided by a large (small) error standard deviation or, indeed, an infinite number of difference-standard deviation combinations. So, one must correct for this confound before rescaling BWS values, which can be done in the same way that one calculates willingness to pay from a DCE [18]. That is, one can divide *k*-1 utility estimates by the *k*-th estimate to render the estimates scale-free; in economics the *k*-th estimate is the value of money (the payment attribute) so dividing the value of a given attribute level by that of money estimates the respondent's willingness to pay.

The paragraph above explains why estimates from *at least *two states must be used to rescale the BWS values. However, it makes sense to choose *only *two states that are close to death to maximise the number of respondents who potentially will consider states to be worse than death. The reasons for this are twofold. First, the value of relatively attractive states would be estimated very imprecisely (due to few respondents choosing death for states high on the latent scale). Second, differences in mean values can only be correctly calculated after adjusting for differences in variances on the latent scale [19]: as stated above, variances are likely to increase and then decrease with improvements in quality of life so states should be picked that lie relatively close together. So, the estimates of states 22222 and 11111 relative to death were used to do this. Whilst state 11112 (for instance) is closer to death than 22222, rescaling the data by a single attribute level estimate would sacrifice information on the other four attributes. The estimate of state 22222 relative to death divided by that of state 11111 relative to death is scale-free; this ratio was used to rescale the BWS values so that zero represents the death state (rather than zero representing state 11111 as in Coast *et al *[11]). The statistical model is set out more formally below.

### Rescaling to ensure death has zero utility

For individual *i *the utility of state *j *over that of state 11111 is given by:

(1) $$U_{ij}=\alpha D_{j}+\vec{\beta }\vec{X_{j}}+\varepsilon_{ij}$$

$\vec{X_{j}}$ is a vector of (5 × 3 = 15) dummy variables, each one representing (for quality of life state *j*) the additional utility of the level of a given attribute over that of level 1 and $\vec{\beta }$ is the vector of coefficients of the dummy variables. *D*_*j *_is a dummy variable equal to 1 for the death state, 0 otherwise. Only the estimated coefficient on the death dummy variable ($\hat{\alpha }$) and estimated coefficients on the five dummy variables representing the additional utility of level 2 over level 1 for each attribute are of interest (State 11111 has zero utility in the model which is estimated in Stata using the *clogit *command.) Thus, *tariff*_22222_, the value of state 22222, is equal to the sum of the five associated elements of $\vec{\beta }$. Then, *ratio*, the base-case scale-free utility of state 22222 relative to state 11111 is:

(2) $$\frac{ta\hat{r}iff_{22222}-\hat{\alpha }}{ta\hat{r}iff_{11111}-\hat{\alpha }}=ratio$$

Solving for $\hat{\alpha }$:

(3) $$\frac{ta\hat{r}iff_{22222}-ratio\cdot ta\hat{r}iff_{11111}}{1-ratio}=\hat{\alpha }$$

Substituting $ta\hat{r}if{f^{\prime }}_{11111}$ and $ta\hat{r}if{f^{\prime }}_{22222}$ (the estimated values from the BWS valuation exercise) into (3) along with the value of *ratio *from the DCE gives ${\hat{\alpha }}^{\prime }$ (the estimated value for death in the BWS model).

Following the rescaling procedure in Coast *et al *[11] of subtracting 1/5 of *tariff*_*death *_from all the BWS attribute level utilities ensured that the 'bottom' anchor (here, death) had zero utility. Dividing by the resulting value of state 44444 ensured that the 'top' anchor (44444) had utility of one. This model assumes all respondents conform to conventional random utility theory in making choices between living states and death. However, as is discussed next, there are reasons to suspect that this may be incorrect in the context of the death state.

### Decision-making processes used by respondents when considering death

Consider a case where a respondent makes a statement along the lines of "life is always worth living". This implies that for that respondent no quality of life state is worse than (or even equal to) death. Thus, those who consider all life worth living, choose between living and death states deterministically instead of stochastically: the probability of choosing a living state as preferred is identically equal to one. Under the random utility model, it can be shown that the probability of choosing a living state over the death state is one if and only if the difference in utility between every living state and death is infinite (see appendix for mathematical proof). Thus, in this case, if the living state is assigned a finite value then the death state cannot be assigned any finite value on the latent continuum. Moreover, if one uses conditional logit to model these choices, one implicitly assumes that these people form the right tail of the error distribution. Yet, no distribution exists for them, and the parameter estimate for '11111 minus death' on the latent utility scale is determined by the relative proportions of people who do and do not trade with death. To show the sensitivity of rescaled values to these proportions, they were varied systematically in simulations using a modified bootstrap procedure [20].

### Sensitivity analysis

To show how a random utility model can result in misleading inferences, two hypothetical types of people were considered: 1) people who make all choices stochastically; and 2) people consistent with the conventional random utility model when trading-off attributes of quality of life, but who are deterministic when comparing states with death (that is, their choice rule is that life is always preferred to death). The proportion of people of each type was varied in a series of analyses by resampling from each of the two types: those who traded with death and those who never traded with death. It should be noted that increasing the proportion of people who traded with death relaxes the assumption that all people in ICECAP observed not to trade would *never *have traded (and were therefore type 2). Respondents were (re)sampled (with replacement) from the actual samples observed in ICECAP such that the observed frequencies of people willing to consider states as worse than death varied from 10% to 75%. The overall sample size was fixed at that number with complete data in the DCE (282). 50 bootstrap resamples were used for each set of proportions; additional resamples are not required to estimate a bootstrap mean compared with estimating, say, a percentile confidence interval around a mean. The mean of the 50 resamples was calculated for the death dummy variable, which represented the estimated utility of death relative to the omitted state (11111), confounded with the unobserved error variance. The sum of the five dummy variables (one for level 2 of each attribute) represents the utility estimate for state 22222 relative to state 11111, again confounded with the unobserved error variance. Equations (1) and (3) were used to estimate the position of death on the BWS scale, enabling rescaled values to be constructed, which make the same assumptions as those in QALYs.

## Results

Data were collected between October 2005 and January 2006, with 478 individuals sampled. This yielded 315 (66%) fully productive interviews (respondents reached the end of the interview), of which 282 answered all 16 DCE comparisons with death. Of the 282 respondents with complete DCE data, 73 (25.6%) considered at least one state to be worse than death. The figures for survey versions A and B were 46 of 151 (30.5%) and 27 of 131 (20.6%) respectively. The higher version A percent likely reflects the fact that state 11111 only appears in A; the worst possible state in version B (state 41111 according to the BWS values) had "attachment" with a better level than 1.

### ICECAP base-case rescaled values

Equation (1) is reproduced below:

(1a) $$U_{ij}=\alpha D_{j}+\vec{\beta }\vec{X_{j}}+\varepsilon_{ij}$$

From the DCE results, the coefficient on the death dummy variable ($\hat{\alpha }$) was estimated to be -0.993. The estimated value of state 22222 (*tariff*_22222_) was 2.942, calculated as the sum of the estimates of the relevant five elements of $\vec{\beta }$. Therefore, on the latent quality of life continuum, using equation (2) to calculate the distance (ratio) with respect to the death state, state 22222 was (2.942 – -0.993)/(0 – -0.993) = 3.964 times as far from death as state 11111. Thus, after 're-anchoring' to incorporate death, the BWS data should *also *have the property that state 22222 is 3.964 times as far from death as state 11111 is. Applying the linear transformation in Coast *et al *[11] to the raw BWS estimates (given in Table 2) but using this additional constraint produces the rescaled estimates, given in the final column of the table. Thus, they are anchored such that death is zero, state 44444 ('full' quality of life) is one, and state 22222 (0.626) is 3.964 times as far from death (0) as state 11111 (0.158).

**Table 2:** Best-worst scaling (BWS), discrete choice experiment (DCE) and rescaled BWS (rBWS) estimates

**State**		**BWS**	**DCE**	**rBWS**
Death state		-	-0.993	0
'No capabilities state' (11111)		0	-	0.158
'Some capabilities state' (22222)		0.556	2.942	0.626

**Attribute**	Level*			

Attachment	4	0.254	1.731	0.245
	3	0.233	0.931	0.227
	2	0.134	1.313	0.144
	1	-0.013	-	0.021

Security	4	0.179	0.264	0.182
	3	0.107	0.479	0.122
	2	0.066	0.143	0.087
	1	0.032	-	0.059

Role	4	0.192	0.472	0.193
	3	0.179	0.551	0.183
	2	0.130	0.385	0.141
	1	0.015	-	0.044

Enjoyment	4	0.166	0.419	0.171
	3	0.164	0.619	0.170
	2	0.119	0.453	0.131
	1	0.017	-	0.046

Control	4	0.209	1.631	0.208
	3	0.185	1.137	0.187
	2	0.108	0.648	0.122
	1	-0.051	-	-0.012

* Numbers refer to levels from most attractive (4) to least attractive (1) and correspond to level wording given in Table 1

Table 2 shows that the DCE estimates are not always rationally ordered for level 2 and above for certain attributes. Indeed, the only estimates that monotonically increase with levels are for "control", which reflects poor precision due to small numbers who consider living states as worse than death. However, it should be noted that these results are used only to illustrate the problem of using limited dependent variable models to anchor living states to the death state; ICECAP was never meant to be a QALY measure. As expected, the estimate of the death state is negative because on average it was considered worse than state 11111 (the omitted state in the DCE); only 26% of people were willing to consider at least one living state to be not worth living – that is, 26% were definitely probabilistic in making choices involving death. Thus, on the latent utility scale state, 22222 is (2.942 – -0.993)/(0 – -0.993) = 3.964 times as far from death as state 11111. The rescaling used ensures that the new BWS values in the final column retain this relationship between states 22222 and 11111.

74% of respondents never considered a living state to be worse than death. However, some of these may change their mind on another occasion, or if different quality of life states had been presented. 39 respondents (14%) are recorded as having spontaneously made statements along the lines of "life is always worth living" (often that is was "God-given") which shows them clearly to be type 2, making choices between living and death states deterministically, not stochastically. There may have been others who made similar comments not recorded by the interviewer and as respondents were not directly asked this question, this 14% can be regarded as the minimum proportion with this view. Therefore, the effect of varying the percentage of people trading with death was investigated in the sensitivity analyses.

### Sensitivity analysis

Table 3 presents seven sets of rescaled values. The first represents the base case state in Table 1 using the actual DCE data from the ICECAP valuation survey. Each of the other six sets presents:

**Table 3:** Sensitivity analysis results

**Set**		**Base**	1*	2**	**3**	**4**	**5**	**6**
Respondents considering states worse than death			10%	25%	30%	40%	50%	75%
'No capabilities state' (11111)		0.158	0.260	0.159	0.128	0.063	0.007	-0.132
'Some capabilities state' (22222)		0.626	0.671	0.627	0.613	0.584	0.559	0.497

**Attribute**	**Level**							

Attachment	4	0.245	0.240	0.245	0.247	0.250	0.253	0.260
	3	0.227	0.224	0.227	0.228	0.230	0.232	0.237
	2	0.144	0.151	0.145	0.142	0.138	0.134	0.125
	1	0.021	0.043	0.021	0.014	0.001	-0.011	-0.041

Security	4	0.182	0.184	0.182	0.182	0.180	0.179	0.176
	3	0.122	0.131	0.122	0.119	0.113	0.108	0.095
	2	0.087	0.101	0.087	0.083	0.075	0.067	0.048
	1	0.059	0.076	0.059	0.054	0.043	0.033	0.010

Role	4	0.193	0.194	0.194	0.193	0.193	0.192	0.191
	3	0.183	0.185	0.183	0.182	0.181	0.179	0.177
	2	0.141	0.148	0.141	0.139	0.134	0.130	0.120
	1	0.044	0.063	0.045	0.039	0.027	0.016	-0.009

Enjoyment	4	0.171	0.175	0.171	0.170	0.168	0.166	0.162
	3	0.170	0.174	0.170	0.169	0.167	0.165	0.160
	2	0.131	0.140	0.131	0.129	0.124	0.119	0.108
	1	0.046	0.064	0.046	0.040	0.028	0.018	-0.007

Control	4	0.208	0.207	0.208	0.208	0.209	0.209	0.211
	3	0.187	0.189	0.187	0.187	0.186	0.185	0.183
	2	0.122	0.132	0.122	0.119	0.113	0.108	0.095
	1	-0.012	0.014	-0.011	-0.019	-0.035	-0.050	-0.084

* Based on only three bootstrap samples: maximum likelihood estimation would not converge on global maximum on iteration 2, 3, or 4 for 8 different seeds for random number generator

** Three bootstrap sets abandoned due to failure to converge on an iteration. Fourth seed chosen successfully allowed a set with 50 bootstrap iterations to be estimated

• Rescaled BWS value for state 11111;

• Rescaled BWS value for state 22222 (calculated from the sum of the five level 2 dummy variables); and

• Best-Worst values rescaled using the DCE estimates according to the model presented above.

Each set of results represents the mean of 50 stratified bootstrap replications using sampling probabilities to produce the frequencies in Table 1.

Table 3 shows that as the percent of people considering states worse than death grows (type 1), most of the rescaled BWS values are 'stretched' downwards. The exceptions are very highly valued attribute levels. Since, for the best possible quality of life states, the 'less valued' attributes of these states are 'stretched downwards' the 'more valued' attributes must be stretched upwards to satisfy the mathematical constraint that state 44444 must have a value of unity. Estimated values for unattractive states become less positive, with state 11111 being zero when the estimated coefficient for the death dummy equals zero, implying that the odds of choosing death over any living state is approximately one. As the proportion of type 1 people grows larger than 50%, the estimated value of state 11111 becomes negative. Thus, for set 6, which has percentages of traders and non-traders approximately in reverse to what was observed in reality, the mean estimate of 11111 is highly negative. This implies that on average people consider this state to be worse than death.

## Discussion

The fundamental limitation of using the ordinal tasks proposed by Salomon and McCabe *et al *[2,3] to anchor living states to death is as follows. Only the TTO or SG (at least conceptually) estimate the trade-off that respondents are willing to make between quantity and quality of life. The estimate of the 'death state' in the ordinal models does *not *conceptually represent this trade-off: it represents the mean distance between the death state and the worst possible living state on the latent (health) scale (confounded with variance scale factor). If any respondents do not conform to the assumptions of conventional random utility theory then this mean is calculated across some values that are infinite and even if all do conform, it is yet to be proven mathematically that this distance conceptually is the same as that trade-off. It is important to note that people who refuse to consider a living state to be worse than death may still consider an impaired health state to be 'worth' a fraction of full health in standard gamble or time trade-off tasks; a person doesn't have to consider states that occur for sure, in other words are *certain*, to be worse (or better) than death to be indifferent between the *lives/gambles *involving impaired health. A given log odds ratio in the McCabe *et al *[3] model cannot be interpreted as a mean difference in latent utility. Instead, it is an average (with unknown weights) of (at least) two groups of people:

• For one group, there is a distribution of utility differences between 11111 and death. Choices of people in this group conform to RUT with death somewhere on the utility scale (other than minus infinity), and may conceivably vary in repeated samples.

• A second group whose choices involving death are deterministic because they consider all life to be worth living – effectively, for members of this group the utility of death is minus infinity.

The reason why the weights are unknown is that respondents who never trade with death in a given valuation exercise may have traded on another occasion or when faced with a worse health/quality of life state. Furthermore, even if the weights were known, one cannot estimate the 'true' population average TTO or SG value because the TTO/SG values for non-traders cannot be recovered in the analysis. The latter point is most obvious when a sample contains only people who always consider life worth living, no matter how bad. The odds that such people will choose death is exactly zero (because crucially, this is *not *a sampling zero), and the utility difference is infinite for a RUT model with EV1 errors (see the mathematical Appendix). Thus, the model proposed by Salomon and by McCabe *et al *cannot be estimated because no-one will choose death, leading to lack of identification of the death dummy variable. In turn, this will lead to all estimates being measured with respect to a remaining state such as 11111. If a given DCE (or ranking/BWS) model purports to estimate values reflecting quantity-quality trade-offs like QALYs, it also must apply to people who never consider health states to be worse than death. The model of Salomon and McCabe *et al *clearly does not satisfy these requirements [2,3].

### Limitations

The true proportion of people unwilling to consider any ICECAP state to be worse than death in the valuation sample was unknown. It is unlikely that the 74% who considered all states worth living were all type 2 people, so some of them might with repeated sampling decide that death was better than 11111 (at least, and possibly other states higher on the latent continuum). It also may be that 11111 was insufficiently unattractive for them to choose death and/or there may be states imaginable to them for which death would be preferable. Nevertheless, as state 11111 was described as having 'none' of any of the five (intended as, in some sense, 'fundamental') attributes of quality of life, it seems reasonable that most of these respondents cannot conceive of a living state that is both worse than 11111 and worse than death.

Potential variation in rescaled values was shown, but the actual degree of bias (deviation from TTO/SG values) could not be calculated as TTO or SG questions were not asked. The ICECAP study will be repeated with a general population sample that will include at least one TTO question to inform this issue. More generally, questions such as "does this model produce estimates with an acceptably low bias?" will in any case require a TTO/SG estimate of state 11111 (or another state), in which case using a RUM to anchor estimates becomes moot.

Although the RUM used is the same as McCabe *et al *and Salomon [2,3], model estimation required more complex analysis to synthesise two different choice processes. Therefore the approach adopted here leads to less clear inferences. However, the approach is justified due to the cognitive burden that would have been imposed by a traditional DCE and the imprecision in model estimates resulting from comparisons with death.

As the percentage of people willing to choose death decreased, estimation issues increased because the estimates became very sensitive to the choices of fewer people. This caused problems for bootstrapping because a given bootstrap sample was likely to include only people with the same preferences, leading to boundary solutions and a failure of maximum likelihood estimation to converge on a global maximum.

### Comparisons with previous work

McCabe *et al*'s model yields an estimated difference between the lowest health state and death that implies an odds of 2.0375:1 for these two states [3]. Thus, approximately 2/3 of people thought the living state preferable to death. Whilst it is possible that greater religiosity led to the higher percentage of non-traders in ICECAP, it has been observed that older people are *more *likely to consider very bad states to be worse than death in EQ-5D data [21]. The ICECAP team's use of best-worst methods to estimate the quality of life values is supported by one of McCabe *et al*'s findings, namely that the latter's models were sensitive only to upper and lower rankings. However, it should be noted that neither Salomon nor McCabe *et al *seemed to consider differences in error variances: in the presence of larger variances around middle rankings the assumption of constant variance artificially reduces the sensitivity of results to these.

Salomon found that the TTO value for the worst possible state was not the same as that from a MNL (random utility) ranking model [2]. This is unsurprising as the estimates will only coincide by chance, and a different proportion of people refusing to consider any states as worse than death may have produced answers that agreed with the TTO estimate.

### Future work

The TTO and SG methods require people to choose between health states lasting for defined periods of time. One can argue that respondents in DCEs (ranking and BWS) should also do the same; that is, respondents would choose a complete health description (lasting for a given length of time) that they prefer. Thus, researchers should consider including length of life as an experimental factor in future preference elicitation studies that use ordinal response tasks. Such an approach requires a more complex study design that is beyond the scope of this paper but guidelines for such designs are now readily available [6].

Future work that estimates QALY values should ensure that statistical models used to analyse data are consistent with decision-making processes (models) used by people. McCabe *et al *state "research on the thought processes of individuals undertaking ranking exercises would be a valuable contribution to this field" [3]. In fact, this understates the seriousness of the issue; we clearly need research to ascertain under what circumstances and to what extent choices satisfy conventional RUT assumptions. Indeed, it seems reasonable to think that a respondent may conform to conventional RUT in one context (comparing quality of life), but not in another (comparing life with death). It also may be that there is no (easy) way to avoid asking one or more TTO/SG questions to properly estimate the anchor at death, and one of Salomon's proposed methods did exactly this [2]. In this event, one should try to minimise the context effects for which some TTO tasks previously were criticised [22]. DCE models incorporating length of life as a variable also deserve investigation.

Philosophical and psychological issues around aggregation of preferences over people who consider bad states worse than death and those who consider all life worth living are pertinent. Recent work suggests growing realisation that this needs more thought before another large QALY valuation exercise is conducted [23,24].

## Conclusion

Conditional logit estimates of utility differences between a given living state and death can be heavily influenced by the proportion of people who consider a state to be worse than death. It does not, and cannot, take into account the utility values of living compared to death for people who make choices involving death deterministically. Moreover, the greater the number of such people in a given DCE, the more biased the estimate of the utility difference because the (assumed) logistic distribution is not defined for people who make choices deterministically instead of stochastically.

## Competing interests

The authors declare that they have no competing interests.

## Authors' contributions

TNF contributed to study design, carried out all data analyses and drafted the paper. AAJM provided substantial conceptual input, wrote the mathematical proof (Appendix) and participated in redrafting of the paper. JJL designed the methodology, advised on study design and analysis and participated in redrafting of the paper. JC participated in the design and running of the study, and in redrafting of the paper. TJP contributed statistical input to the study and participated in redrafting of the paper. All authors read and approved the final manuscript.

## Appendix

### Summary

If the extreme value random utility version of the (conditional) multinomial logistic (MNL) model is assumed to hold and the choices of a respondent are such that *P*(*L*, *D*) is identically (not statistically) equal to 1 for all living states *L *≠ *D*, where D is death, then u(D) cannot be assigned any finite value, such as 0.

Let L denote a typical quality of state, i.e. a living state, and D death. Let *P*(*L*, *D*) denote the probability that an individual respondent chooses L over D. Assume that the respondent's choices satisfy the random utility version of MNL. That is, there is a (standard) extreme value (type 1) random variable *ε *such that (Pr(*ε *≤ *t*) = exp(-*e*^-*t*^); (∞ <*t *< ∞), and scale values *u*(*L*) and *u*(*D*) such that

*P*(*L*, *D*) = Pr(*u*(*L*) + *ε *> *u*(*D*) + *ε*')

where *ε *and *ε*' are independent samples of the random variable. Then classic results show that

(4) $$P(L,D)=\frac{e^{u(L)}}{e^{u(L)}+e^{u(D)}}=\frac{e^{u(L)-u(D)}}{e^{u(L)-u(D)}+1}$$

### Case 1: When is P(L, D) ≡ 1 for all living states L ≠ D?

(1) implies this is the case if, and only if, for all *L *≠ *D*, *u*(*L*) - *u*(*D*) = ∞. The only possible solutions are:

i) for all *L *≠ *D*, *u*(*L*) = ∞, *u*(*D*) < ∞,

ii) for all *L *≠ *D*, *u*(*L*) > -∞, *u*(*D*) = -∞.

#### Comment 1

In case 1i) we would expect the respondent to choose randomly between any two living states, *L*_1 _and *L*_2_, provided neither was death (though that choice probability would not be given by the RUM MNL as it can be interpreted as given by

$$P(L_{1},L_{2})=\mathrm{Pr}(\infty +\varepsilon >\infty +\varepsilon ')=\frac{\infty }{\infty +\infty },$$

which is undefined. In any case such choices do not seem to be made in real data. So we are left with case 1ii), where the utilities of different living states *L*_1_, *L*_2_, neither being death, can (but do not have to) differ, and all such states are chosen deterministically (i.e. with probability one) over death.

#### Comment 2

We can fit the MNL to the data of a respondent satisfying Case 1ii, but we cannot use death as a 'referent' state that is assigned some finite value, for example, 0, as the true value of death is -∞.

### Case 2: When is P(L, D) > 1 - *δ*, with *δ *'small', for all living states L ≠ D?

Using (1), routine algebra shows that this holds provided that, for all *L *≠ *D*,

$$u(L)-u(D)>\ln\frac{1-\delta }{\delta }.$$

However, as *δ *approaches zero, the right hand side approaches infinity, and thus,

for all *L *≠ *D*, *u*(*L*) - *u*(*D*) approaches ∞ which brings us back, effectively, to Case 1.

### Conclusion

If the extreme value random utility version of the MNL model is assumed to hold and the choices of a respondent are such that *P*(*L*, *D*) is identically (not statistically) equal to 1 for all living states *L *≠ *D*, where D is death, then *u*(*D*) cannot be assigned any finite value, such as 0.
